# Perceptions, attitude and use of family planning services in post conflict Gulu district, northern Uganda

**DOI:** 10.1186/s13031-015-0050-9

**Published:** 2015-08-11

**Authors:** Christopher Garimoi Orach, George Otim, Juliet Faith Aporomon, Richard Amone, Stephen Acellam Okello, Beatrice Odongkara, Henry Komakech

**Affiliations:** Department of Community Health and Behavioural Sciences, Makerere University-School of Public Health, P.O Box 7072, Kampala, Uganda; St. Marys Hospital Lacor, 180, Juba Road, Gulu, Uganda; Uganda Bureau of Statistics, 7186, Plot 9 Colville Street, Kampala, Uganda; International Health Sciences University, 7782, St. Barnabas Road, Kampala, Uganda; Kyambogo University, Kampala, Uganda; Gulu University, Faculty of Medicine, Kampala, Uganda

**Keywords:** Family planning services, Accessibility, Attitudes, Perceptions, Utilization, Post conflict, Northern Uganda

## Abstract

**Background:**

Northern Uganda was severely affected by two decades of civil war that led to the displacement and encampment of an estimated 1.6 million inhabitants. The objective of this study was to assess community perspectives, attitude and factors that influence use of family planning (FP) services in post conflict Gulu district.

**Methods:**

We conducted a cross sectional study using multistage sampling technique. All three counties in the district were purposely selected. Two sub-counties per county and four parishes per sub-county were randomly selected. A total of 24 parishes (clusters) and 21 adult heads of households per cluster were randomly selected and interviewed. In total, 500 adults 117 males (23.4 %) and 383 females (76.6 %) were interviewed. We conducted 8 focus group discussions and 6 key informant interviews with family planning managers and service providers. Quantitative data were entered in EPI data and analyzed using STATA version 12. Qualitative data were analyzed manually using thematic content analysis.

**Results:**

Contraceptive prevalence rate was 47.5 %. Communities perceive FP as acceptable, beneficial and geographically, temporally and financially accessible. Factors associated with FP use included age 26–35 years (AOR 1.92, 95 % CI 1.18-3.10, *p* = 0.008), and 36–45 years (AOR 2.27, 95 % CI 1.21-4.25, *p* = 0.010), rural residence (AOR = 0.41, 95 % CI 0.24-0.71, *p* = 0.001), cohabitation (AOR = 2.77, 95 % CI 1.15-6.65, *p* = 0.023), and being a farmer (AOR 0.59, 95 % CI 0.35-0.97, *p* = 0.037). The main reason for non-use of family planning was fear of side effects 88.2 %. The main source of FP services was government health facilities 94.2 %.

**Conclusion:**

Use of family planning is relatively high and communities view FP services as acceptable, beneficial and accessible. Family planning use is mainly determined by age, residence, occupation and marital status. Fear of side effects is the main impediment to FP use. There is need to increase awareness and effectively manage side effects of family planning in the settings.

## Background

During 1986–2006, northern Uganda experienced an intractable civil war that led to the displacement of an estimated 1.6 million (90 %) of the inhabitants into Internally Displaced Persons (IDP) Camps [1]. The civil war led to poor health status with the region having the worst health indices in the country [2]. The total unmet need for family planning in the northern region, among married women was 46 % compared with 41 % nationally. Contraceptive use was at 12 % compared to the national average of 23 % [3]. There was high rate of abortions and unwanted pregnancies. One in every five pregnant women in northern Uganda had had an abortion, while 50 % of pregnancies were unwanted [2]. The total fertility rate estimated at 7.5 was one of the highest in the country [4]. Thus, the protracted civil war presented several challenges to the delivery of health services including family planning in the settings [5].

Armed conflicts lead to forced displacement and encampment as well as profound negative impacts on health status including sexual and reproductive health of the affected communities [6]. Encampment is often associated with restricted movements and poor access to basic social services including health [7]. Wars lead to disruption of health systems - destruction of health infrastructure, shortage of human resources for health, scarcity of supplies and equipment and hamper access to health services [8]. Furthermore, wars, displacement and encampment limit access to and the attainment of the right to sexual and reproductive health [9, 10].

The provision of sexual and reproductive health services to displaced populations was for long overlooked in humanitarian emergencies. International and local relief organizations often focus on the provision of basic necessities including food, water and sanitation, shelter and basic health care services to reduce high rates of morbidity and mortality [6]. The 1994 International Conference on Population and Development held in Cairo and the 1995 Fourth World Conference on Women in Beijing brought to light the sexual and reproductive health needs of displaced populations. Subsequently, the international community response has been reoriented and includes the provision of comprehensive sexual and reproductive health services to displaced populations including family planning, gender-based violence, and sexually transmitted infections (STIs) including HIV/AIDS [11].

Studies on family planning use in post conflict settings are limited. A study of family planning perspectives of couples in post conflict settings in Democratic Republic of Congo revealed that inadequate knowledge, poor service provision, male dominance, and intimate partner violence had negative effects on access to and use of family planning services [12]. An evaluation of a programme to expand reproductive health services reported that integration of mobile teams and targeted health systems strengthening made family planning services easily accessible in conflict affected settings [13]. The objective of the study was to examine community perceptions, attitude and factors associated with the utilization of family planning services in post conflict Gulu district, northern Uganda.

## Methods

### Study settings

This study was a community survey conducted in Gulu district, northern Uganda. Gulu is one of 6 post conflict districts in the Acholi sub region that was severely affected by the over two decades of civil war. The war was fought between the Lord’s Resistance Army and the Government of Uganda during 1986–2006. Gulu district has an estimated population of 443,773 inhabitants [14]. The district has a total of 68 health facilities, 51 (75 %) being government and 17 (25 %) belonging to non-governmental organizations. There are 4 hospitals including Gulu Regional Referral hospital, St Mary`s Hospital Lacor, Gulu Independent hospital, and Uganda People Defence Forces Army hospital Lower level health facilities include; 2 Health Centre (HC) level IV, 14 Health Centre level III and 48 Health Centre level II in the district.

### Data collection procedure

We conducted a cross sectional study between December 2013 and February 2014. We used both quantitative and qualitative techniques of data collection. The study population consisted of adult heads of household, women aged between 18–49 years, and men 18–60 years who had lived in the district for more than six months.

Gulu district has three counties. We purposely selected all three into the study. Two sub-counties per county were randomly selected from each county. From each of the two selected sub-counties, four parishes were randomly sampled. Hence a total of 24 parishes were randomly selected from the entire district. A parish formed the study cluster.

We used the World Health Organization (WHO) modified cluster sampling methodology to select the study respondents [15]. In each cluster, the centre of the cluster was identified. To determine the initial direction of movement, a bottle was spun. The initial direction was determined by the direction pointed by the mouth of the bottle. We determined the number of households from the centre to the end of the cluster. The starting household was determined by listing all household from the centre to the end of the cluster in the predetermined direction. The starting household was then randomly selected. After the starting household, every second nearest household was then selected and a respondent interviewed. Thus, we selected and interviewed the study respondents in the ratio of 1 male to 3 females. In each parish (cluster), we interviewed a total of 21 adult heads of households. Hence a total of 117 males (23.4 %) and 383 (76.6 %) females were interviewed.

The community survey data were collected using a structured questionnaire administered by research assistants who had been trained for two days. Quantitative data were collected on participant’s demographics, perceptions, attitude, use and availability of family planning services. The questionnaire was developed in English, translated into the local language, Acholi and then back translated into English. The questionnaires were pretested in two parishes that were not included in the final list of clusters for data collection.

Qualitative data were collected using both Focus Group Discussions (FGDs) and Key Informant Interviews (KII). Focus groups discussions were conducted with women and men aged between 18–49 years and 18–60 years respectively in different counties and parishes. The FGDs were conducted using a semi-structured topic guide. The topic guide addressed perceptions, attitude and factors related with family planning use in the post-conflict era. The FGD participants were identified from among the survey participants with the help of the community leaders, and purposefully selected. A total of 8 FGDs, two per selected group of women, men, adolescents and community leaders comprising local councilors (LCs) were conducted. The group discussions were moderated by trained researchers who spoke the local language-Acholi.

We conducted 6 key informant interviews. The KIIs were conducted with the district family planning focal person, in-charges of health units, managers, and family planning service providers in public and private not for profit health facilities. The KII covered several themes including service availability, access to family planning and service provision. The provider questionnaire addressed issues relating to the availability of family planning methods, logistics, provider attitude and beliefs, family planning demand, and conflict-related issues. Both FGDs and KIIs were tape recorded.

### Data management and analysis

Quantitative data were captured using EPI data version 3. The data were cleaned and analysed using STATA version 12. Descriptive analysis was carried out to explore levels of awareness, knowledge of different types of contraceptive, sources of family planning information and services, attitude and practice among respondents. Bivariate analysis assessed the relationships between selected demographic, socio-economic variables and current contraceptive use. Logistics regression analysis was carried out to determine predictors of family planning use. Statistical significance was considered at *p*-values less than 0.05.

The dependent variable was a dichotomous response variable that was assigned the value 1 if the respondent was using any contraceptive method and 0 if not using any method. Several socio-demographic factors may influence the decision of to use contraceptives. We included significant socio-demographic variables at bivariate analysis with a p value of < 0.05 in the model to determine the association between the dependent and independent variables. Variables used for the analysis included age, education, religion, marital status, residence and occupation.

Qualitative data from the FGDs and KIIs were recorded as sound files using digital recorders and later transcribed to text files. Transcripts of the recorded discussions were coded and analysed manually using thematic content analysis and participants’ identification details were removed. Codes and categories that emerged from data were sorted out to form the main themes. To ensure internal consistency and reliability, both quantitative and qualitative results were triangulated.

### Ethical considerations

Ethical clearance for the study was obtained from the Higher Degrees and Research Ethics Committee of Makerere University School of Public Health and Uganda National Council of Science and Technology. Permission to conduct the study was obtained from the District Health Officer, Gulu district. Informed consent was sought and obtained from all study participants. All data were kept confidential. Respondent names were excluded from the recorded materials to avoid disclosure of the identity of the participants.

## Results

### Socio-demographic characteristic of respondents

As shown in Table 1 below, most respondents 376 (75.2 %) were aged between 18–35 years. The vast majority 376 (98.2 %), were Christians with 335 (67.0 %) being Catholics. More than half 296 (59.2 %), had attained primary education, a fifth 111 (22.2 %) had secondary level education and 54 (10 %) had no formal education. Most respondents 366 (73.2 %) resided in rural areas.

**Table 1:** Socio-demographic characteristics of respondents

Characteristics	Sex				Total	
	Males (117)		Females (383)		N (500)	
	n	%	n	%	n	%
Age in Years						
18–25	25	21.4	118	30.8	143	28.6
26–35	59	50.4	174	45.4	233	46.6
36–45	18	15.4	75	19.6	93	18.6
>46	15	12.8	16	4.2	31	6.2
Religion						
Catholic	77	65.8	258	67.4	335	67.0
Protestant	30	25.6	71	18.5	101	20.2
Muslim	1	0.9	7	1.8	8	1.6
Pentecostal	9	7.7	47	12.3	58	11.2
Education Level						
None	1	0.9	53	13.8	54	10.8
Primary	57	48.7	239	62.4	296	59.2
Secondary	44	37.6	67	17.5	111	22.2
College/University	15	12.8	24	6.3	39	7.8
Marital Status						
Single	12	10.2	21	5.5	33	6.6
Married	56	47.9	146	38.1	202	40.4
Separated	3	2.6	29	7.6	32	6.4
Widow/widower	1	0.8	26	6.8	27	5.4
Cohabiting	45	38.5	161	42.0	206	41.2
Occupation						
Unemployed	16	13.7	137	35.8	153	30.6
Farmer	54	46.2	175	45.7	229	45.8
Business	21	17.9	52	13.6	73	14.6
Civil servant	14	11.9	9	2.3	23	4.6
Private/NGO	12	10.3	10	2.6	22	4.4
Residence						
Urban	31	26.5	103	26.9	134	26.8
Rural	86	73.5	280	73.1	366	73.2

### Awareness, use and sources of family planning information and services

As illustrated in Table 2, the study shows that nearly half, 182 (47.5 %) of the female respondents were currently using modern contraceptives. The majority 273 (71.3 %) of the females in this study had ever used modern contraceptives. Most respondents 375 (77 %), reported discussing contraceptive use with their spouses.

**Table 2:** Awareness, use and sources of family planning information and services

Variables	Sex				Total	
	Males		Females			
	n	%	n	%	n	%
Use of FP methods						
Current use of contraceptive	50	42.7	182	47.5	232	46.4
Ever used contraceptive	90	76.9	273	71.3	363	72.6
Discuss contraceptive use with spouse	95	81.2	290	75.7	375	77.0
Decision to use FP						
Both	48	71.6	142	71.0	190	71.1
Self	15	22.4	38	19.0	53	19.9
Spouse	4	6.0	20	10.0	24	9.0
Awareness of contraceptive methods ^a^						
Male condoms	84	71.8	117	30.5	201	40.2
Female condoms	8	6.8	37	9.7	45	9.0
IUD	36	9.4	187	48.8	223	44.6
Oral pills	71	60.7	288	75.2	359	71.8
Implants	61	52.1	276	72.1	337	67.4
Vasectomy	16	13.7	35	9.1	51	10.2
Tubal legation	12	10.3	75	19.6	87	17.5
Injectable	16	13.7	41	10.7	57	11.4
Sources of FP information ^a^						
Government facility	86	73.5	302	79.1	387	77.4
Media	44	37.6	96	25.1	140	28.0
Peers/colleagues	11	9.4	50	13.1	61	12.2
Private health facility	14	12.0	33	13.1	47	9.4
Family planning outreach	14	12.0	31	8.6	45	9.0
Pharmacy/drug shop	4	3.4	4	8.1	12	2.4
Community based distributor	3	2.6	6	1.6	9	1.8
Sources of FP services ^a^						
Government/public facility	98	83.3	373	97.4	471	94.2
Private facility	23	19.6	74	19.3	97	19.4
Family planning outreach	6	5.1	12	3.1	18	3.6
Peer/colleagues	1	0.9	13	3.4	14	2.8
Pharmacy/drug shop	7	6.0	4	1.0	11	2.2
Community based distributor	0	0.0	3	0.8	3	0.6

*IUD* intrauterine contraceptive device, *FP* family planning,

^a^ Multiple responses were provided

Among those using family planning services, most respondents 190 (71.1 %) stated joint decision making regarding use of family planning. Joint decision making among couples was corroborated in the male FGD as shown in the excerpt below:

*“In my household, I and my wife decided that we should stop having children ..... we agreed to have 5 children and once we realized that number we decided to stop”****(FGD male, 36–46 years).***

Findings indicate that participants held the view that, men as heads of households were the decision makers. As such men decided on family planning use. As decision-makers, men were expected to initiate discussions on family planning use. Women were considered as implementers of the decisions made, without questioning men’s decisions. This view is illustrated in the female FGD excerpts below;

*“Men are very reluctant to discuss FP. They think there is no need to. The husband will tell the wife the number of children they are to have, and that is it. It is the man who decides. The woman does not have any say and cannot oppose anything that has been decided upon by the man; she has less control and does as the man instructs”****(FGD female, 26–35 years).***

Most respondents 387 (77 %) obtained family planning information from public health facilities, and 140 (28 %) from the media. The vast majority of the respondents 471 (94.2 %) received family planning services from public health facilities while 97 (19.4 %) from private facilities. The qualitative findings also indicated that public health facilities are the main sources of family planning services;

*……….Except for the drug shops in urban areas, the public health facilities are the main providers of such services****(KII, health worker).***

There were mixed attitude towards use of family planning in the post conflict era. The feeling of having lost children during the conflict encouraged the community to produce more children in the post conflict period. This was supported by findings from the male FGD;

*“There are some households who lost all their children and these (people) see family planning as a bad thing. They intend to produce children to replace those who died during the war”****(FGD male, 26–35 years).***

While women were more receptive to family planning use, there were concerns that men perceived it negatively. Males were partly responsible for women not using contraceptives. This finding was supported by men FGD as shown below.

“*The problem is with us men, the women want to use contraceptives but we always refuse them and for that matter some women have resorted to using contraceptives discreetly”****(FGD male, 35–45 years).***

### Distance, reasons for use and non-use and benefits of using family planning

As shown in Table 3; most respondents 415 (72.0 %) resided between 0–5 km of the nearest health facility. The most common reasons for using contraceptives were that it promotes healthy growth of children, 139 (34.5 %), better maternal health 107 (26.5 %) and ensures enough resources for the family 105 (26.1 %). The benefits of family planning were pointed out by respondents in  the female FGDs as illustrated;

*“Family planning is good, because I have seen and experienced the benefits. If you space your children, they will grow up healthy without being sickly most of the time. Ill health that comes as a result of not spacing your child is avoided and as the mother of your children also relaxes from the challenges associated with motherhood”****(FGD female, 26–35 years).***

**Table 3:** Distance, reasons for use and non-use and benefits of family planning services

Variables	Total	
	n	%
Distance to FP Services ^a^		
Less than a km	209	41.8
2–5 km	206	30.2
6 km +	85	17.0
Reason for use of FP Services ^a^		
Children to grow up healthy	139	34.5
Maternal health is better	107	26.5
Enough resources for family	105	26.1
Helping prevent STI/HIV	26	6.5
Preventing pregnancy related risks	17	4.2
Reducing adolescent pregnancies	7	1.7
Reducing infant mortality	2	0.5
Reason for Non use of FP Services ^a^		
Side effects	359	88.2
Irregular period	34	7.7
Natural methods more effective	17	3.9
Some methods don’t protect against HIV	14	3.2
Reduces Sexual pleasure	9	2.0
Adherence to methods not easy	9	2.0
Benefits of FP services ^a^		
Child spacing	200	40.0
Better child health	151	30.2
Enough resources for the family	117	23.4
Preventing unwanted abortions	29	5.8
Preventing related health risk in women	24	4.8
Preventing STI/HIV	19	3.8
Prevent unwanted pregnancy	11	2.2

^a^Multiple responses were provided

The most commonly cited reasons for not using contraceptive were fear of side effects 359 (88.2 %), irregular periods 34 (7.7 %) and the view that natural methods are more effective 17 (3.9 %). This finding was substantiated by the FGDs indicating fear of side effects as a barrier to use of contraception and these included excessive bleeding. This led to discomfort and insecurity among women. This is expounded in the female FGDs below:

*“Like for my case, when the bleeding starts, sometimes it is so heavy that it may go on for up to two weeks and this worried me a lot, it nearly broke up my marriage. Because, if the flow from this month’s period is still going on the next will come and just continue so it is really heavy”****(FGD female, 18–25 years).***

*When I was using the Injectable I did not experience my periods for a long time and that made me worried for so long and once I started experiencing my periods again, it was endless! Because it was flowing endlessly and I was embarrassed to even sit with my fellow women and it also led to a brief separation between me and my husband. I had to return to my parents’ home but later returned.****(FGD female, 18–25 years).***

Concerns over side effects associated with family planning methods were further expressed by key informants. For instance, one service provider stated that:

*Bleeding …. that is the most common side effect I know. It scares people because some women even become anaemic as a result of over bleeding. The other is that many women complain of nausea and this has been reported widely along with weight loss, loss of appetite among many others****(KII health worker, female).***

Similar views were expressed by women that the side effects are not easily managed at health facilities. Individuals have to meet the costs of treating side effects when they seek services from private health service providers.

*I can go to access these services, but then when it comes to managing the side effects, if you go back to the health centre to remove it - the FP method, for cases of implants, in most cases they often refuse to remove it*……… *it then becomes very costly when you have to treat yourself for these side effects****(FGD female, 18–25 years).***

Religion was identified as a barrier to family planning use. A provider with knowledge of family planning but with religious convictions may not be willing to provide family planning services. During the KIIs, some respondents pointed out the role of religion as a barrier to family planning.

*The level of knowledge and religious affiliation of the service provider also play key role. For example our midwife here at the health centre is a Catholic and for her she will never give any method of family planning to anyone whenever she is on duty****(KII female, health worker).***

Findings from men and women FGDs also indicate that religious belief was a reason for non-use of family planning as pointed out in the male FGD:

*“Let me say this, as a Catholic, our religion does not allow us to use contraceptives so that alone can prevent us from using contraceptives”****(FGD male, 26–35 years).***

Spousal opposition to family planning encourages covert use of family planning by women. If the husband objects to contraceptive use, women cannot freely use contraceptives because of the repercussions if discovered. This view was expressed in several female FGDs;

*“If my husband is against me using contraceptives, I have no option but make my own decision. For some method once I have been injected with the contraceptive, I just go home and my husband will never know.”****(FGD female, 26–35 years).***

*“Sometimes I even fear that my husband will find out the FP method I am using and that will bring quarrels. So I cannot think of using it”****(FGD female, 26–35 years).***

The main benefits of using family planning included child spacing 200 (40 %), better child health 151 (30.2 %), and having enough resources for the family 117 (23.4 %).

### Suggestions towards improving accessibility and utilization of family planning services

As shown in Table 4, the main suggestions made towards improving accessibility of family planning services were to ensure availability of family planning providers 167 (33.4 %), carrying out sensitization 159 (31.8 %) and ensuring availability of family planning supplies. The findings from KIIs were in agreement as illustrated by the excerpt below:

*“Looking at this Health Centre of ours…the situation still needs a lot of improvement because, the health workers are few and on several occasions when clients come to facility they are either turned away or told that there is no medicine”****(KII female, health worker).***

**Table 4:** Suggestions towards improving accessibility and utilization of family planning services

Variables	Sex				Total	
	Male		Female			
	n	%	n	%	N	%
Suggestions towards improvement of accessibility						
Ensure availability of health providers	37	31.6	130	33.9	167	33.4
Sensitization	37	31.6	122	31.8	159	31.8
Ensure availability of FP supplies	24	20.5	74	19.3	98	19.6
Ensure availability of facilities	13	11.1	41	10.7	54	10.8
Affordability of services	6	5.1	16	4.3	22	4.4
Suggestions towards improvement of utilization of FP services						
Sensitization	86	73.5	247	64.5	333	66.6
Ensure availability of supplies	15	12.8	76	19.8	91	18.2
Improve skills of FP providers	10	8.5	23	6.0	33	6.6
Availability of FP providers	5	4.3	23	6.0	28	5.6
Affordability	1	0.8	14	3.7	15	3.0

The main suggestions made towards improving utilisation of family planning services included ensuring sensitization of the community members 333 (66.6 %), ensuring availability of family planning supplies 91(18.2 %), and improving the attitude and skills of providers 33 (6.6 %). This finding was substantiated with findings from female FGDs;

*“Sometimes I believe FP methods are available, but the right personnel are the problem especially when it concerns IUDs not all the health workers know how to insert it. The health workers therefore need more training regarding the application of family planning methods”****(FGD female, 26–35 years).***

### Logistic regression analysis of factors associated with use of contraceptives

As shown in Table 5, the main factors that are significantly associated with the use of family planning were age 26–35 years AOR 1.92, 95 % CI 1.18-3.10, *p* = 0.008, 36–45 years AOR 2.27, 95 % 1.21-4.25, *p* = 0.010 residence in rural area AOR 0.41, 95 % CI 0.24–0.71, *p* = 0.001, and cohabitation AOR 2.77, 95 % CI 1.15-6.65, *p* = 0.023, occupation-farmer AOR 0.59, 95 % CI 0.35-0.97, *p* = 0.037.

**Table 5:** Logistic regression analysis of factors associated with use of contraceptives

Variable	COR	(95 % CI)	*P* value	AOR	(95 % CI)	*P* value
Age						
18–25 ^a^	1.00			1.00		
26–35	2.11	1.37–3.25	0.000	1.92	1.18–3.10	0.008*
36–45	1.89	1.10–3.23	0.018	2.27	1.21–4.25	0.010*
>46	0.38	0.15–0.96	0.032	0.50	0.18–1.14	0.192
Sex						
Male ^a^	1.00			1.00		
Female	0.82	0.54–1.25	0.364	0.76	0.46–1.25	0.278
Residence						
Urban †	1.00			1.00		
Rural	0.33	0.21–0.52	0.000	0.41	0.24–0.71	0.001*
Marital status						
Single †	1.00			1.00		
Married	3.73	1.65–8.45	0.000	3.33	1.35–8.22	0.001*
Divorced/separated	0.90	0.31–2.65	0.848	0.70	0.21–2.28	0.009*
Widow/widower	0.52	0.15–1.81	0.298	0.54	0.14–2.11	0.553
Cohabiting	3.15	1.40–7.06	0.000	2.77	1.15–6.65	0.023*
Occupation						
Unemployed ^a^	1.00			1.00		
Farmer	0.53	0.34–0.80	0.025	0.59	0.35–0.97	0.037*
Business	1.23	0.69–2.22	0.471	0.93	0.48–1.810	0.836
Civil servant	0.84	0.34–2.04	0.697	0.59	0.21–1.71	0.335
Private/NGO	0.64	0.26–1.59	0.336	0.50	0.18–1.43	0.198

Variables included in AOR; age, sex, residence, marital status, and occupation

*Significant at 5 % level

^a^ Reference category

## Discussion

The study has shown that 4 in 10 adults are currently using family planning in post conflict Gulu district, northern Uganda and the attitude towards family planning is that it`s acceptable, beneficial and is geographically, temporally and financially accessible. The main factors associated with use of family planning include age, residence, marital status and the occupation of respondents. However, fear of side effects is a major impediment to use of family planning services.

Our finding revealed a higher use of family planning services than the national average of 42 % [16]. The main sources of family planning information and services were public health facilities. The plausible reasons for the high use of family planning services may include the fact that access to health facilities is high and the vast majority of health services (67 %) in the district are rendered by public health facilities. Furthermore, several Non-Governmental Organizations (NGOs) have been operating in the district both during and in the post conflict periods supporting health services provision including family planning programmes. The involvement of the various NGOs ensured the availability of health personnel, contraceptive supplies and commodities. In addition, community sensitization alongside the involvement of the district and village health teams could have been responsible for the increased uptake of family planning services by the communities. Moreover, the study also revealed that there is positive community attitude towards family planning. This may be attributed to the extensive sensitization and awareness creation campaign activities carried out by several humanitarian relief organizations working in the district.

The results show that respondent’s age was associated with contraceptive use among older women compared to the younger ones. This was expected, as older women are more knowledgeable and keener on their fertility preference [17]. This finding is in agreement with that of a study conducted in Uganda which revealed that age is a significant predictor of contraceptive use [18]. Our study revealed that respondents who were living with a partner or cohabiting use more family planning services. This finding is consistent with that of a study conducted in South Africa which revealed that living with a partner or cohabitation is associated with contraceptive use [19]; and a study in Uganda also reported that rural residents were less likely to use contraceptives compared with urban residents [20].

The main impediment to use of family planning, identified by this study was concern about the side effects of contraceptives. The Uganda Demographic Health Survey findings of 2011 also identified fear of side effects among the leadings reasons for non-use and discontinuation of contraceptive use [21]. Women were concerned about the side-effects and the costs associated with their management. Contraceptive use raised issues related to social risks and difficulties in marriage and relationships owing to the side effects of the methods. Negative experiences and misconceptions about particular types of contraceptives undermine uptake of family planning services. This has been reported in Nepal and Ethiopia [22–24]. It is imperative that key stakeholders, institutions and health care providers make concerted efforts to address the side effects associated with the various types of contraceptives as well as provide adequate information to the public about the management of the side effects.

Our study revealed that most respondents discuss family planning use with their spouses.

The decision to use FP was made in most cases (71 %) by both men and women, and only in 1 out of 5 cases (19.9 %) by self (i.e. 24 % men and 19 % women) respectively, however, several female focus group discussions indicated that men often made the final decision about family planning use. The finding of this study is similar to that of a previous study in northern Uganda by Natabbi et al. which revealed that males dominate decision making around fertility issues [25].

Men’s influence regarding fertility, maternal and child health services has been documented in many traditional patriarchal societies [26]. Gender relations play an important role in decision-making and are an essential aspect of the social context of reproductive health [27]. Previous studies in Uganda and Ethiopia have also shown that male dominated decision making about fertility preference is associated with lower use of contraceptives [28, 29]. This suggests the need for continued sensitization and empowerment of women towards decision making in the area of sexual and reproductive health.

In our study, we found that women resorted to covert use of contraceptives. This may be attributed to differences in gender relations and lack of communication between couples. Similar findings have been reported in Tanzania where women were found to be using contraceptives secretly [27]. Covert use of family planning exposes women to several risks including financial, emotional and physical violence if discovered by their spouses. Our study also revealed that if men discover that their wives are using contraceptives, they are forced to have them removed. Thus, women often had to return to family planning service providers to have the implants removed. In this study woman who returned to the facility reported that some health workers refused to remove the implants because it was not yet time to remove them despite the pressure from their spouses. Similar findings have been reported in Nigeria [30]. Providers are indifferent when women returned prematurely to have the implants removed and they often declined. This has a negative effect on adoption and continued use of long term acting contraceptives. Women are less likely to adopt and use such family planning options without support and approval of their spouses. This therefore calls for increase in family planning counselling of couples by providers in similarly affected settings.

Several suggestions were made towards improving access to and utilization of family planning services in the setting. These included ensuring that skilled health care providers are available in adequate numbers, supplies and equipment are readily available and that community sensitization is adequately carried out. These suggestions indicate that the support of key stakeholders including the various implementing agencies and international NGOs rendered towards the district health services delivery is critical to improving sexual and reproductive health services including family planning. It is acknowledged that family planning brings large potential health and survival benefits for children and mothers owing to wider birth intervals and aversion of obstetric related morbidity and mortality [31]. Thus, the support of the various partners towards effective FP services delivery contributes to the improvement of maternal, neonatal and child health outcomes in the settings.

Our study had limitations. It was conducted in only one of the six post conflict districts in the Acholi sub-region, northern Uganda. This was due to financial limitation. Hence this may limit the generalization of the study to the wider post-conflict northern region. However, the Acholi sub-region is mainly inhabited by one major ethnic tribe, the Acholi, and prior to decentralization it was only one district, Gulu. Thus Gulu district may be largely representative of the rest of the districts in the Acholi sub-region. In addition, although we had planned that a third of the heads of households to be interviewed be males, owing to the difficulty of getting male heads of households at home during the period of the survey, the proportion was reduced to 23.5 %.

### Conclusions and recommendations

The study has shown that use of family planning is relatively higher in the post conflict Gulu district compared to the national average and the perception is that family planning services are accessible, beneficial and should be embraced by community members. The main factors influencing use of family planning include age, residence, occupation and marital status. However, fear of side effects is the most important reason for non-use of contraceptives. The various stakeholders involved in the implementation of family planning programmes should therefore intensify awareness campaigns on specific methods of contraception and the management of possible side effects, and improve public, private and outreach family planning services provision to improve uptake and reproductive health outcomes.
